# Supplementary material for: Testosterone is an endogenous regulator of BAFF and splenic B cell number
Source: Nat Commun. 2018 May 25;9:2067. doi: 10.1038/s41467-018-04408-0 (PMC5970247; doi:10.1038/s41467-018-04408-0)
Supplement: Supplementary file 1 — Supplementary Information [file 41467_2018_4408_MOESM1_ESM.pdf]

SUPPLEMENTARY INFORMATION

Testosterone is an endogenous regulator of BAFF and splenic B cell number

Wilhelmson et al.

SUPPLEMENTARY FIGURES

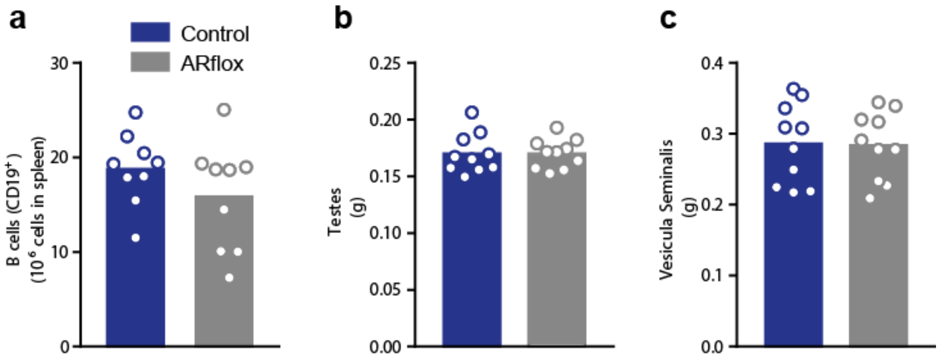

Supplementary Figure 1. Number of splenic B cells and weights of reproductive organs in ARflox mice. (**a**) Number of $CD19^{+}$ B cells in spleen from control (Ar^{+}) and Ar^{flox} male mice; n = 9/group. (**b-c**) Weight of testes (**b**) and seminal vesicles (**c**) in control (Ar^{+}) and Ar^{flox} male mice; n = 10/group. All bars indicate means; circles represent individual mice.

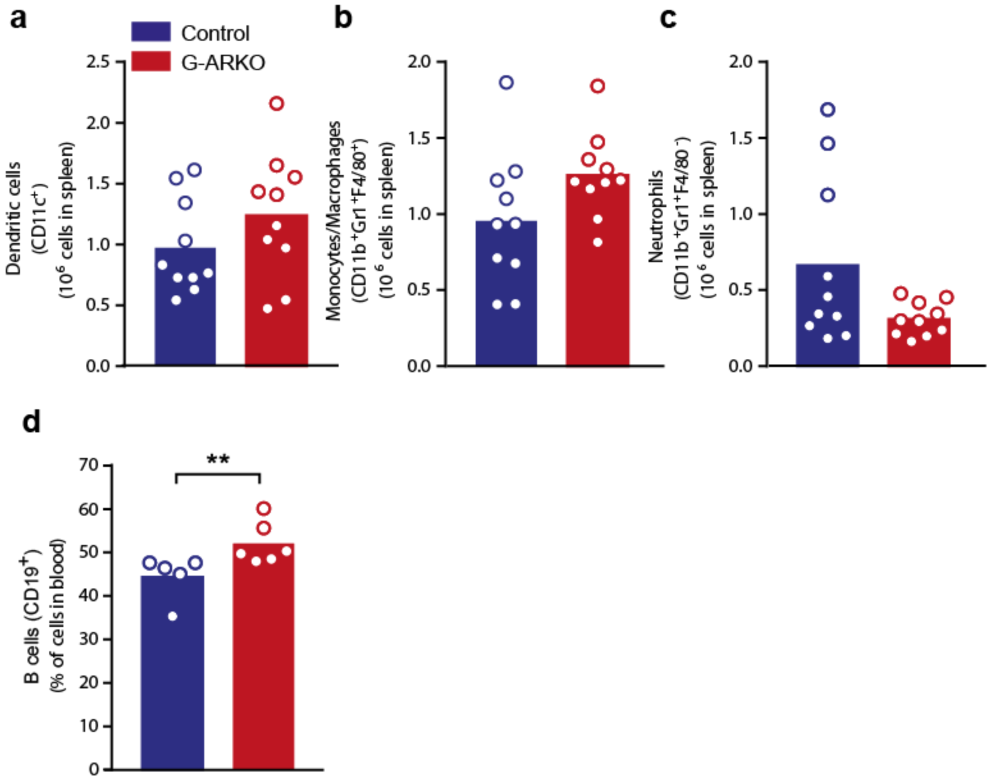

Supplementary Figure 2. Immune cells in G-ARKO mice. (**a**) Number of $CD11c^{+}$ dendritic cells, (**b**) $CD11b^{+}Gr1^{+}F4/80^{+}$ monocytes/macrophages, and (**c**) $CD11b^{+}Gr1^{+}F4/80^{-}$ neutrophils in spleen from control (*Pgk-Cre*$^{+}$) and general androgen receptor knockout (G-ARKO) male mice. n = 10/group. (**d**) Percent $CD19^{+}$ B cells in blood from control (n = 5) and G-ARKO (n = 6) male mice. All bars indicate means; circles represent individual mice.$^{**}P < 0.01$ (Mann-Whitney test).

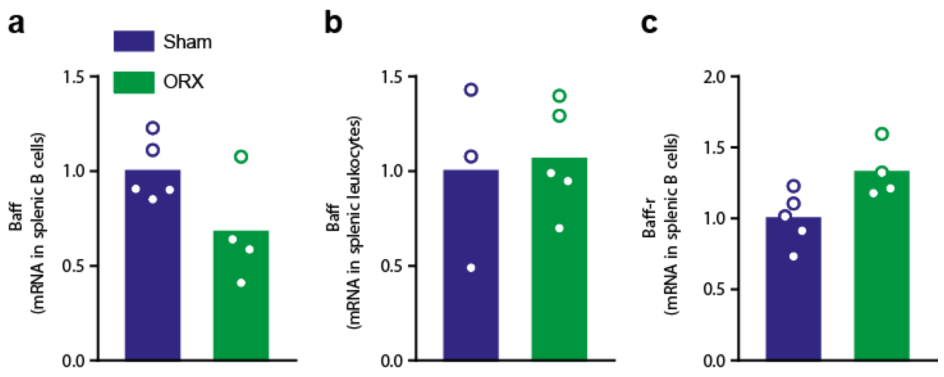

Supplementary Figure 3. The effect of castration on splenic *Baff* and *Baff-r* mRNA. (**a-b**) *Baff* mRNA in sorted splenic CD19$^+$ B cells (**a**) and other (CD19$^-$CD45$^+$) splenic leukocytes (**b**) from sham-operated (*n* = 5 and 3, respectively) and castrated (ORX; *n* = 4 and 5, respectively) male mice. (**c**) *Baff-r* mRNA levels in sorted splenic B cells from sham-operated controls (*n* = 5) and castrated (*n* = 4) male mice. All bars indicate means; circles represent individual mice.

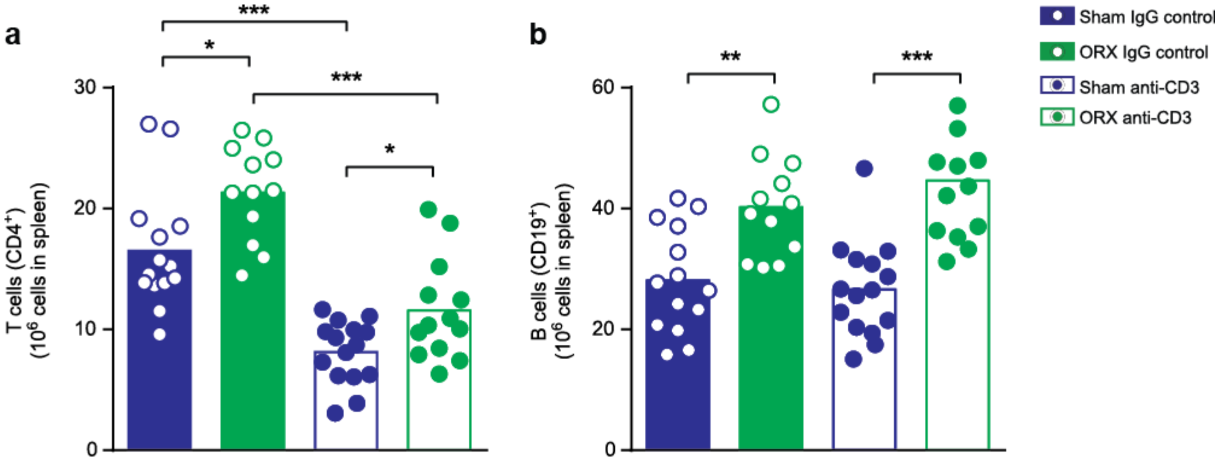

Supplementary Figure 4. Antibody-mediated T cell depletion does not alter the effect of castration on splenic B cell number. (**a**) T cells ($CD4^+$) and (**b**) B cells ($CD19^+$) in spleen in control IgG-treated and anti-CD3-treated sham-operated or castrated (ORX) male mice at 16 weeks of age; 2 weeks after last antibody injection. Surgery was performed at 4 weeks of age and antibody injections given from 5 weeks of age, with 3-week intervals. n = 12–15/group. All bars indicate means; circles represent individual mice. $*P < 0.05$, $**P < 0.01$, $***P < 0.001$ (Kruskal-Wallis followed by Mann-Whitney test).

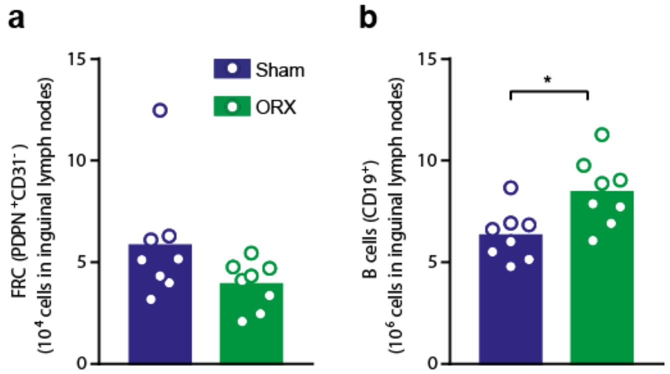

Supplementary Figure 5. The effect of castration on numbers of FRCs and B cells in lymph nodes. Number of (**a**) fibroblastic reticular cells (FRCs; $CD45^{-}PDPN^{+}CD31^{-}$) and (**b**) B cells ($CD19^{+}$) in inguinal lymph nodes from sham-operated and castrated (ORX) male mice. n = 8/group. All bars indicate means; circles represent individual mice. $^{*}P < 0.05$ (Mann-Whitney test).

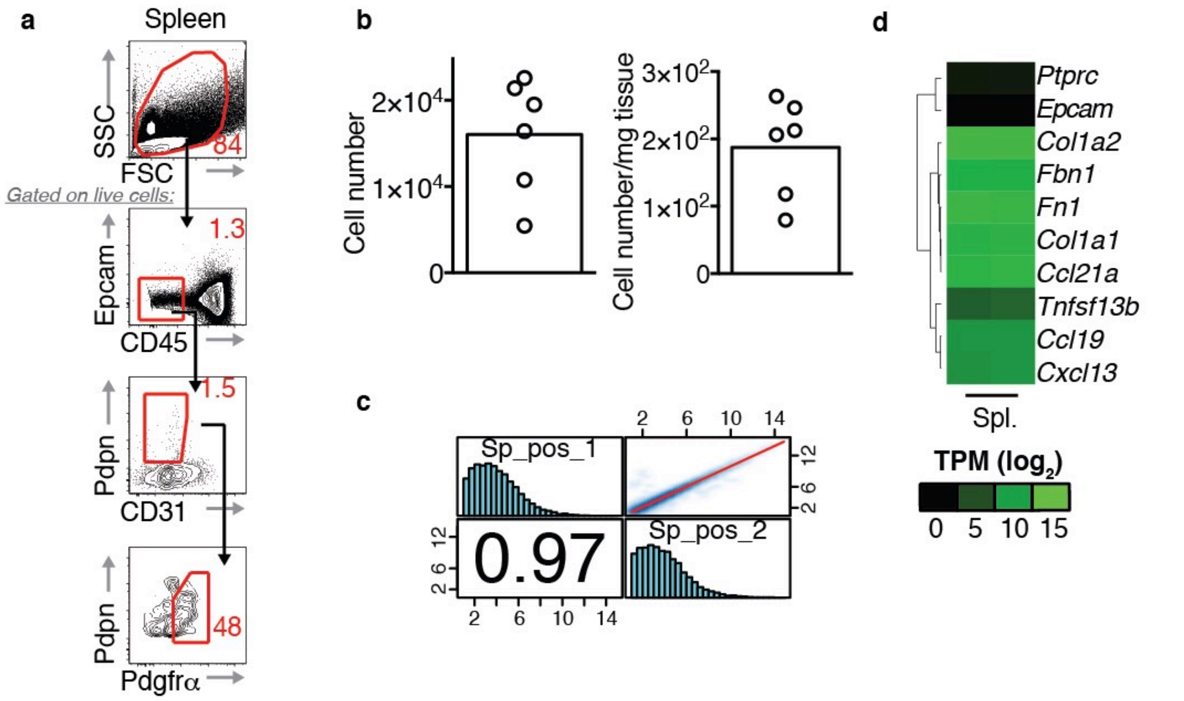

Supplementary Figure 6. Gene expression in splenic FRCs. (**a**) Gating strategy and representative flow cytometry plots used to sort splenic fibroblastic reticular cells (FRC); numbers in red denote frequency of gated cells. (**b**) Number of live, $CD45^{-}CD31^{-}PDPN^{+}$ FRCs from spleen and number of FRCs per milligram of tissue. Data are from two experiments. Each dot represents one mouse. (**c**) Similarity matrix between RNAseq samples. Lowly expressed genes were removed (below 1 transcripts per million, TPM) and (TPM + 1) values were then log2 transformed. Pairwise Spearman correlation statistics were calculated among all samples and reported here (lower left). (**d**) Expression of fibroblast and FRC genes in sorted splenic FRC. Corresponding data on adrenergic receptor expression presented in Fig. 6a.

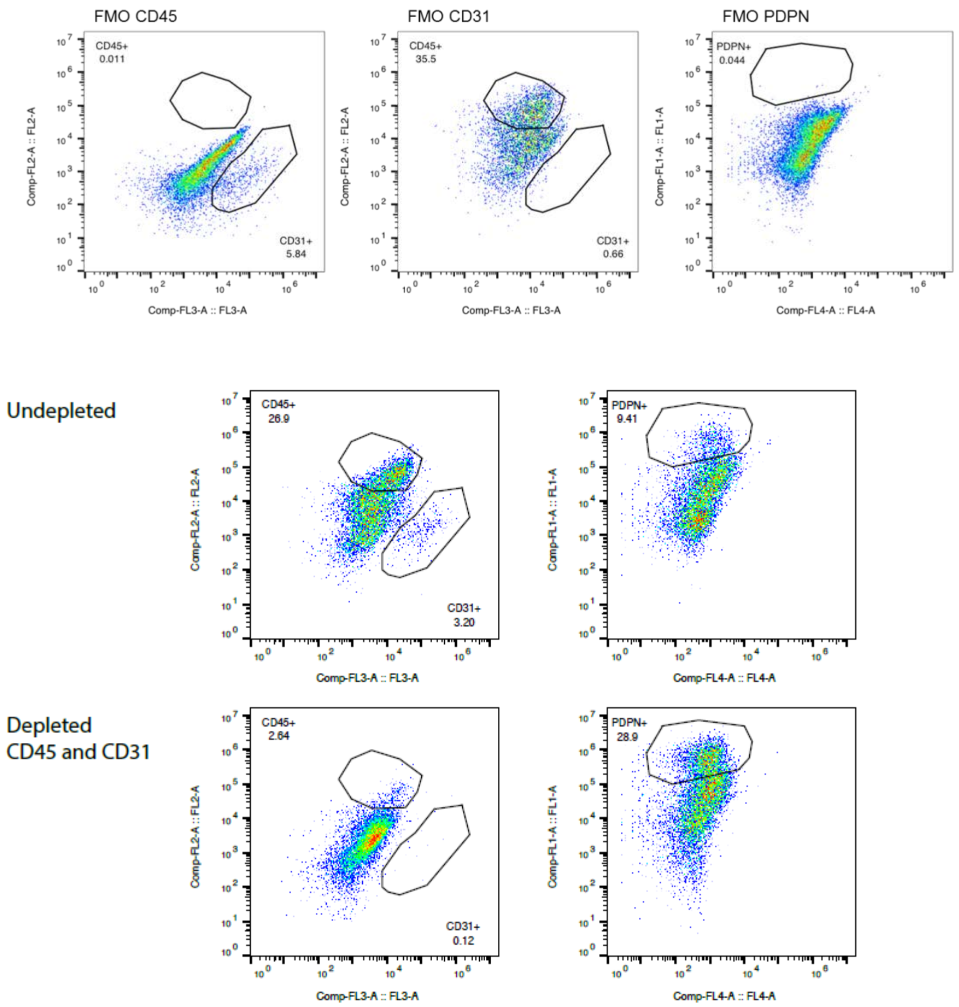

Supplementary Figure 7. Splenic fibroblastic reticular cell (FRC) culture *in vitro*. Spleens from wild-type male mice were digested and after culture for 6 days, cells were enriched for nonhematopoietic and nonendothelial cells with anti-CD31 and anti-CD45 microbeads. The stromal cell composition was assessed by flow cytometry for CD31, CD45, and PDPN before and after enrichment.

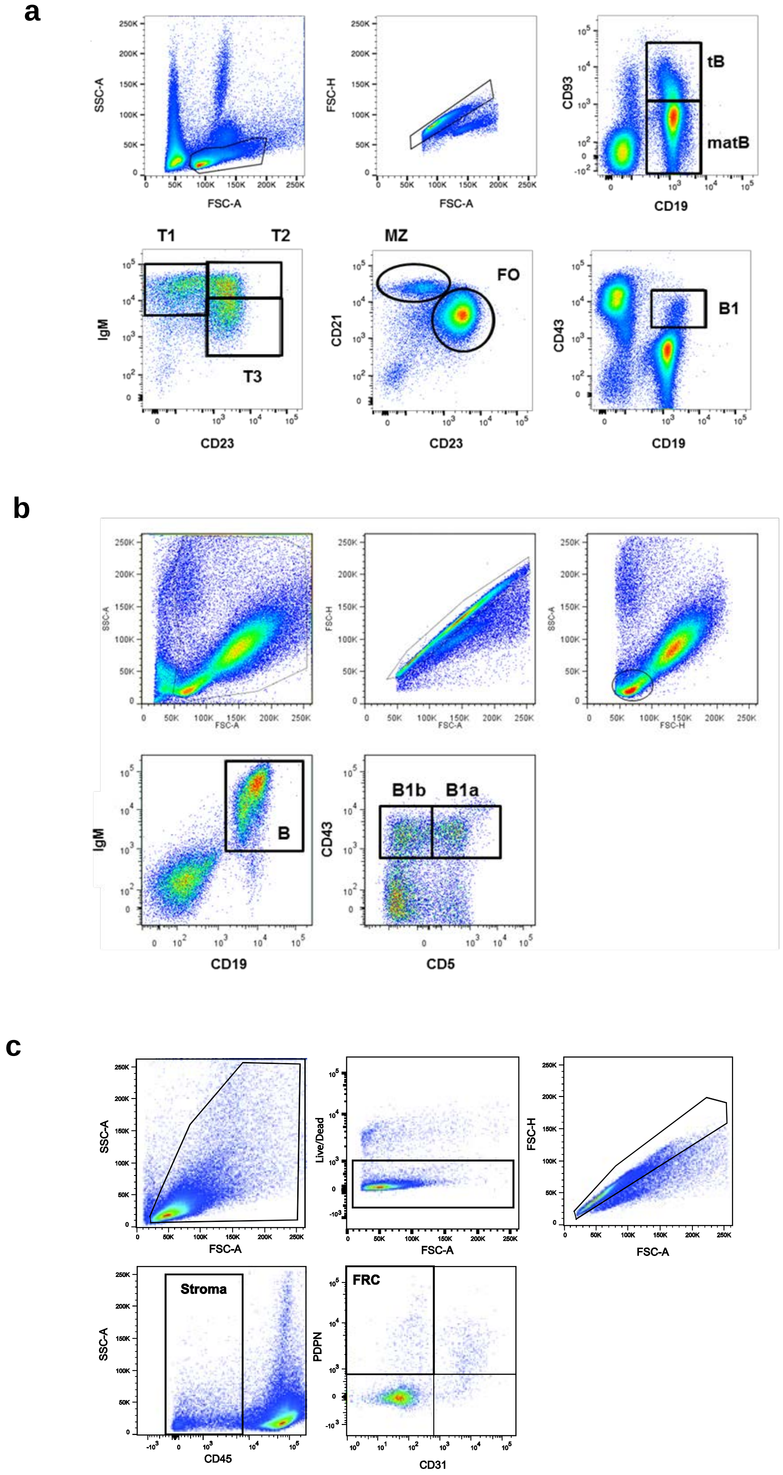

Supplementary Figure 8. Gating strategies. (**a**) Splenic B cells (data in Fig. 1b-c, 1e-j, 2a-d, 3h-k, 6h). Nucleated cells were gated to exclude red blood cells; smaller fragments and nonsingle cells were excluded. Transitional B cells (tB) were identified as $CD19^{+}CD93^{+}$ and mature B cells (matB) as $CD19^{+}CD93^{–}$. These were further divided into T1–3 cells by IgM and CD23 expression and follicular (FO) and marginal zone (MZ) B cells by CD21 and CD23 expression. B1 cells were identified as $CD19^{+}CD43^{+}$. (**b**) Peritoneal B cells (data in Fig. 1d). Nucleated cells were gated to exclude red blood cells; smaller fragments and nonsingle cells were excluded. Lymphocytes were selected and gated for IgM. $CD19^{+}$ B cells were identified as $CD19^{+}IgM^{+}$, B1a cells as $CD43^{+}CD5^{+}$, and B1b cells as $CD43^{+}CD5^{-}$. (**c**) Splenic FRCs (data in Fig. 5d-f, 6b). Smaller fragments, dead cells, and nonsingle cells were excluded. Stromal cells were identified as $CD45^{–}$ and of these FRCs (fibroblastic reticular cells) were identified as $PDPN^{+}CD31^{–}$.

SUPPLEMENTARY TABLES

Supplementary Table 1. Characteristics of eugonadal and hypogonadal men

**Characteristics**	**Eugonadal (*n* =114)**	**Hypogonadal (*n* = 12)**	***P* value**
Age (years)	36 ± 8	42 ± 8	0.006
Body mass index (kg/m^2)	25 ± 3	27 ± 4	0.022
Total testosterone (nmol/L)	19 ± 5	10 ± 6	<0.0001
Free testosterone (nmol/L)	0.38 ± 0.08	0.18 ± 0.03	<0.0001

Values are means ± s.d. P-values are from t-test.

Supplementary Table 2. Primers used for genotyping

Gene	Type	Primer sequence (5’-3’)
Ar (exon 2 locus)	F	AGCCTGTATACTCAGTTGGGG
	R	AATGCATCACATTAAGTTGATACC
Cre	F	AACATGCTTCATCGTCGG
	R	TTCGGATCATCAGCTACACC
Zfy	F	AAGATAAGCTTACATAATCACATGGA
	R	CCTATGAAATCCTTTGCTGCACATGT

We assessed *Ar*[1] and *Cre* genotypes using PCR amplification of genomic DNA. Male sex (presence of the Y chromosome) was confirmed by detection of the *Zfy* gene[1].

REFRERENCES

1. De Gendt, K., *et al.* A Sertoli cell-selective knockout of the androgen receptor causes spermatogenic arrest in meiosis. *Proc Natl Acad Sci U S A* **101**, 1327-1332 (2004).
